# Identification of Novel Centenarian Phenotypes From the Kuakini Honolulu Asia Aging Study: A Latent Class Analysis

**DOI:** 10.1093/geroni/igaf122.068

**Published:** 2025-12-31

**Authors:** Yeonjung (Jane) Lee, Gina Lee, Peter Martin, Kamal Masaki, Bradley Willcox

**Affiliations:** University of Hawaiʻi at Mānoa, Honolulu, Hawaii, United States; University of Wisconsin–Madison, Madison, Wisconsin, United States; Iowa State University, Ames, Iowa, United States; Kuakini Medical Center, Honolulu, Hawaii, United States; John A. Burns School of Medicine, Honolulu, Hawaii, United States

## Abstract

As society ages, understanding the determinants of healthy aging and longevity has become a research priority. Most studies of longevity lack prospectively collected data. Therefore, we utilized the Kuakini Honolulu Asia Aging Study (HAAS), a decades-long, prospective epidemiologic study of aging. Based on known predictors of longevity, including education, parents’ history of Alzheimer’s disease, stress management, social support, family longevity, family networks, FOXO3 and APOE genotypes. We performed a latent class analysis in Mplus and identified three novel, distinct centenarian phenotypes (subgroups). One, “socially buffered” survivors (16.20% of the sample) had a relatively higher level of education and social support, despite the lowest mother’s age at death. Two, the “longevity guardians” (5.63% of the sample) had the greatest number of children, the highest proportion of healthy longevity FOXO3 gene, and the highest levels of stress tolerance but the lowest levels of education. The “academic” longevity group (78.17% of the sample) had the highest mother’s age at death and highest levels of education, despite the lowest number of children. In terms of functional ability, the socially buffered survivors had the highest ADL impairment level, whereas the longevity guardians had the lowest impairment level. Our study advances the understanding of different pathways to longevity, based on family longevity, social support and network, stress management, educational attainment, and genetic factors among Japanese-American male centenarians. The findings provide implications for continued investigation on lifestyle and genetic factors promoting healthy aging and offer insights into the evidence on the strengths and resilience of long-lived individuals.

